# Enhancing Expressway Traffic State Perception: A Novel BAS-Optimized PSO-BP Fusion Model with Tensor Completion

**DOI:** 10.3390/s26102998

**Published:** 2026-05-10

**Authors:** Jiacheng Yin, Xiaofei Guo, Wei Bai, Lijing Ma, Li Tang

**Affiliations:** 1 School of Automobile and Transportation, Xihua University, Chengdu 610039, China; yinjiacheng@xhu.edu.cn (J.Y.); guoxiaofei@stu.xhu.edu.cn (X.G.); malijing@mail.xhu.edu.cn (L.M.); tangli@xhu.edu.cn (L.T.); 2 Department of Transportation Management, Sichuan Police College, Luzhou 646000, China; 3 Intelligent Policing Key Laboratory of Sichuan Province, Luzhou 646000, China

**Keywords:** expressway, multi-source data fusion, BSO-BP neural network, traffic flow parameter estimation

## Abstract

With the continuous expansion of the expressway network and the rapid growth of traffic demand, traditional single-source traffic detection data is limited in spatial–temporal coverage and accuracy, which can hardly support the refined operation and management of intelligent expressways. Existing data preprocessing methods often fail to fully capture global spatiotemporal features, and traditional PSO-BP neural networks are prone to local optima. To address these issues, this study investigates multi-source traffic data fusion using ETC-DSRC and RTMS microwave data from the Jiangsu section of the G50 Shanghai-Chongqing Expressway. The HaLRTC tensor completion algorithm is adopted to repair missing and abnormal data, fully mining the spatial–temporal correlation characteristics of traffic flow. The beetle antennae search (BAS) mechanism is introduced into the particle swarm optimization (PSO) process to improve particle search behavior and population diversity. On this basis, a BAS-optimized PSO-BP neural network, referred to as BSO-BP in this study, is constructed for multi-source traffic data fusion. In this model, the improved PSO algorithm is used to optimize the initial weights and thresholds of the backpropagation (BP) neural network, thereby improving the global search capability and convergence stability of the fusion model. Taking the average road speed as the fusion target, MAE, RMSE and MAPE are used for accuracy verification. The results show that the proposed model has significantly higher accuracy than single-source data methods and BP, PSO-BP, and GA-PSO-BP models, and can reflect the real traffic state of road sections more accurately.

## 1. Introduction

Transportation is a strategic, foundational, and service-oriented sector in China’s national economy. As a core component of comprehensive transport systems, expressways play an irreplaceable role in improving efficiency, enabling intensive resource allocation, and promoting regional coordinated development. The continuous expansion of expressway networks and the increasing complexity of their structure have become key characteristics of China’s transport infrastructure [1]. Alongside this expansion, traffic demand has grown rapidly, leading to congestion, frequent accidents, and declining service levels on some sections. Traditional digital management can no longer meet operational needs, making the shift to information-based and intelligent management essential. The Outline for Building a Leading Transportation Nation also calls for deep integration of artificial intelligence, big data, and other emerging technologies into transportation [2] to build advanced, ubiquitous infrastructure. A prerequisite for this goal is the accurate, real-time understanding of traffic flow states through full analysis of traffic big data [3].

Traffic data collection is fundamental to intelligent expressway management. Currently, China’s expressways employ a multi-sensor system combining various technologies. On one hand, traditional fixed-point detectors such as inductive loops and remote traffic microwave sensors (RTMS) are widely used; these provide high-precision, high-frequency cross-sectional parameters (volume, speed, occupancy) but are limited by sparse deployment and susceptibility to data loss due to equipment failure or adverse conditions. On the other hand, the nationwide adoption of Electronic Toll Collection (ETC) enables collection of DSRC data from OBU–RSU interactions, offering a new means of obtaining interval-based traffic states. ETC provides continuous, spatially extensive data but struggles to capture real-time conditions at specific cross-sections and may include errors from faulty OBUs [4]. Additional technologies such as GPS floating cars and mobile phone signaling offer complementary spatial coverage but face limitations from signal occlusion and base station density, making them insufficient alone for refined traffic control [5]. Given the limitations of any single data source, fusing multi-source data to leverage their complementarity has become essential for accurate traffic state representation and for supporting intelligent management.

Multi-source traffic data fusion has become a research focus in traffic engineering and intelligent transportation. In terms of application, fusion techniques are widely used for traffic parameter estimation, incident detection, state prediction, and traffic control. In terms of algorithms, existing research mainly includes methods based on classical statistics/probability (e.g., Bayesian methods, Kalman filtering, D-S evidence) and those based on neural networks. Classical methods are theoretically mature but often rely on restrictive assumptions and have limited ability to handle data uncertainty. Neural networks, with their strong nonlinear fitting and learning capabilities, have become mainstream; the PSO-BP algorithm, combining the global search ability of particle swarm optimization (PSO) with the local fitting of backpropagation (BP), is particularly common in traffic data fusion. However, existing studies still have several limitations. Regarding data sources, most research focuses on loops, microwave detectors, and GPS data, with relatively insufficient use of ETC data [6,7,8,9]. Given the full coverage of ETC on China’s mainline expressways, fusing ETC-DSRC data with conventional detector data holds significant practical value. Regarding algorithm optimization, traditional PSO-BP suffers from insufficient population diversity, susceptibility to local optima, and poor convergence; although some studies have used genetic algorithms for improvement, these issues have not been fundamentally resolved [10]. Regarding data preprocessing, anomaly detection and imputation are critical for fusion accuracy. Most existing methods rely on time series, spatial interpolation, or historical data, which only use local information and cannot capture global spatiotemporal traffic patterns [11,12]; as a result, imputation accuracy is often insufficient, especially when data are missing over consecutive intervals.

To address these gaps, this study develops a multi-source expressway traffic data fusion framework using ETC-DSRC and RTMS microwave data. The framework consists of three main components: data completion, spatiotemporal matching, and neural-network-based fusion [13,14]. First, leveraging spatiotemporal correlations, the HaLRTC tensor completion method is employed for anomaly imputation, integrating both temporal and spatial information to improve accuracy. Second, to overcome the limitations of the traditional PSO-BP algorithm, we propose a BSO-BP fusion algorithm that uses the beetle antennae search (BAS) algorithm to enhance population diversity and convergence, thereby improving fusion accuracy. Finally, a multi-source traffic data fusion model based on BSO-BP is constructed and validated using empirical data from the G50 Shanghai–Chongqing Expressway. The effectiveness and accuracy of the proposed method are demonstrated through comparisons with single-source estimates and results from other fusion algorithms. The findings are intended to advance multi-source expressway traffic data fusion methodologies, enhance the accuracy and reliability of traffic parameter estimation, and provide robust technical support for intelligent expressway operations, traffic control decisions, and public information services.

## 2. Multi-Source Traffic Data Acquisition and Fusion

### 2.1. Multi-Type Traffic Detection Technologies

Expressway traffic detection technologies are primarily classified into two categories: single-point cross-section detection and interval trajectory detection. Based on their detection principles, data characteristics, and engineering applicability, and after comprehensively considering detection accuracy, data continuity, and engineering application scenarios, RTMS microwave detection data and ETC-DSRC detection data are selected as the fusion data sources [15,16].

### 2.2. Data Fusion Technology

The two collection technologies described above generate complementary heterogeneous data sources, characterized by differing data quality and spatiotemporal coverage [17]. If only a single detector type is used for traffic operation analysis and research, incomplete or inaccurate data collected during the data acquisition phase may compromise the estimation or prediction results, casting doubt on the accuracy and certainty of the outcomes. Therefore, fusing data acquired from multiple types of traffic detection technologies has become a critical step in achieving precise traffic parameter estimation or traffic state analysis.

In this paper, a neural network-based approach is selected for multi-source traffic data fusion, and improvements are made to address its susceptibility to becoming trapped in local optima [18,19]. The effectiveness of the fusion model is quantitatively evaluated using three classic indicators: mean absolute error (MAE), root mean square error (RMSE), and mean absolute percentage error (MAPE). These three indicators provide a comprehensive and objective assessment of the accuracy and reliability of the fusion results from three perspectives—absolute error, sensitivity to outliers, and proportion of relative deviation—thereby establishing a unified and standardized evaluation criterion for subsequent model validation and comparative analysis.

## 3. Construction of a Multi-Source Data Fusion Model Based on an Improved PSO-BP Neural Network Algorithm

### 3.1. BAS-Optimized PSO-BP Neural Network Algorithm

In this study, BSO-BP is used to denote a BAS-optimized PSO-BP neural network model. It should be noted that BSO is not treated as an independent optimization algorithm in this paper. Instead, the term BSO-BP describes a combined modeling structure in which the beetle antennae search (BAS) mechanism is embedded into particle swarm optimization (PSO), and the improved PSO is then used to optimize the backpropagation (BP) neural network. Specifically, BAS provides an antennae-based directional search mechanism by comparing the fitness information detected from the left and right search directions of each particle. This mechanism helps improve particle search behavior, maintain population diversity, and reduce the possibility of premature convergence. The BAS-optimized PSO algorithm is subsequently employed to determine the initial weights and thresholds of the BP neural network. Therefore, the logical relationship of the proposed model is BAS → improved PSO → optimized BP, and the resulting fusion model is referred to as BSO-BP.

The particle swarm optimization (PSO) algorithm is simple, involves few parameters, and is easy to implement [20]. However, as seen from the velocity update equation, the particle’s movement direction is jointly influenced by inertia, cognitive, and social parameters, and the trajectory is semi-random. With fixed parameters, selecting appropriate values is crucial for optimization. A large inertia weight ensures search independence in the early stage but leads to slow convergence later. Overly large cognitive and social parameters may cause particles to skip the optimal solution due to excessive step sizes. Moreover, as all particles converge toward the best individual, movement becomes homogenized, reducing population diversity and making the algorithm prone to local optima [10,21]. Therefore, the BAS mechanism is incorporated into PSO in this study to address these shortcomings and to construct a multi-source traffic data fusion model based on the BSO-BP neural network.

In the BSO-BP model, the position and movement direction of each particle are determined not only by its own velocity and the global and individual optimal positions, but also by the directional information perceived through the beetle antennae search mechanism. By introducing this mechanism, the search trajectory of particles can be further diversified, thereby improving the global exploration capability of the optimization process. The optimized particle position represents a candidate set of initial weights and thresholds of the BP neural network, and the fitness value is calculated according to the prediction error of the neural network. Through iterative optimization, the BAS-optimized PSO algorithm searches for the optimal initial parameters of the BP neural network, after which the BP network is trained for multi-source traffic data fusion.

Compared with the conventional PSO-BP model, the substantive difference of the proposed BSO-BP model lies in the particle update mechanism. In PSO-BP, PSO is directly used to optimize the initial weights and thresholds of the BP neural network. The particle position is updated only according to the inertia term, the individual best position, and the global best position. Although this mechanism can accelerate the search process, particles tend to move toward the current optimal individual during iteration, which may reduce population diversity and cause premature convergence.

In contrast, the proposed BSO-BP model introduces the BAS mechanism into the PSO update process. Each particle is assigned a beetle antennae search behavior by constructing left and right antennae along its movement direction. The fitness values detected by the two antennae are compared to determine an additional search increment, which is then incorporated into the particle position update. Therefore, BSO-BP does not simply replace PSO with BAS; rather, it uses BAS as a directional sensing mechanism to enhance the particle search process within PSO. This modification enriches the search trajectory of particles, improves local exploration around candidate solutions, and helps maintain population diversity during iteration. As a result, the proposed model is expected to reduce the risk of local optima and improve the convergence stability of PSO-BP.

The specific algorithm steps are as follows:

1. After determining the neural network structure, the population dimension is calculated. Let *I* be the number of input layer neurons, *H* be the number of hidden layer neurons, and *O* be the number of output layer neurons. The population dimension *D* is calculated as: D=I·H+H+H·O+O.
2. Initialize relevant parameters, including those required for PSO and BAS iterative computations, the population size *N*, and the maximum number of iterations $T_{max}$.
3. Define a fitness function (e.g., root mean square error, mean absolute error, or mean absolute percentage error of the sample data) to evaluate each particle.
4. Introduce the BAS algorithm to equip each particle with beetle antennae search behavior, and determine the coordinates of the left and right antennae:
  (1) $$\left\{\begin{array}{c} x_{ird}^{t}=x_{id}^{t}+v_{id}^{t}\times d^{t}/2\\ x_{ild}^{t}=x_{id}^{t}-v_{id}^{t}\times d^{t}/2 \end{array}\right.$$
  where:
  $x_{ird}^{t}$—coordinate of the *d*-dimensional right antenna of particle *i*;
  $x_{ild}^{t}$—coordinate of the *d*-dimensional left antenna of particle *i*.
  Calculate the increment of the particle’s beetle antennae search behavior:
  (2) $$\xi_{id}^{t+1}=\delta^{t}\times v_{id}^{t}\times sign\left(f\left(x_{ird}^{t}\right)-f\left(x_{ild}^{t}\right)\right)$$
5. Update particle velocity and position:
  (3) $$\left\{\begin{array}{cc} v_{id}^{t+1} & =wv_{id}^{t}+c_{1}\left(pbest_{id}^{t}-x_{id}^{t}\right)R_{1}+c_{2}\left(gbest_{id}^{t}-x_{id}^{t}\right)R_{2}\\ x_{id}^{t+1} & =x_{id}^{t}+\left(1-\lambda \right)v_{id}^{t+1}+\lambda \xi_{id}^{t+1} \end{array}\right.$$
  Compared with the conventional PSO-BP model, the key difference in the proposed BSO-BP model lies in the additional BAS-based search increment $x_{id}^{t+1}$ in the position update equation. This term is generated by comparing the fitness values detected from the left and right antennae of each particle, and it provides additional directional search information for particle movement. Therefore, BSO-BP introduces an antennae-based local exploration mechanism into PSO, which helps maintain population diversity and reduce premature convergence.
6. Repeat the iterative control process. Check whether the target accuracy δ is achieved or the preset maximum number of iterations $T_{max}$ is reached. If not, update the inertia weight according to Formulas (3) and (4). A decreasing formula is used to ensure a larger search space in the early stage for global exploration and refined local search in the later stage.
  (4) $$w=w_{\max}-\frac{w_{\max}-w_{\min}}{T_{\max}}\cdot t$$
  where:
  $w_{\max}$—upper limit of inertia weight;
  $w_{\min}$—lower limit of inertia weight.
7. The optimal particle position obtained by the BAS-optimized PSO process is used as the initial weights and thresholds of the BP neural network.

### 3.2. Multi-Source Traffic Data Fusion Model Based on the BSO-BP Neural Network Algorithm

This paper constructs a BSO-BP neural network fusion model based on BAS-improved PSO and applies it to expressway traffic detection data. The relevant model settings are described below.

The fusion data sources are microwave detectors and ETC toll collection systems, which are fused to obtain more accurate section speed [22]. According to traffic flow theory, speed and flow are correlated; therefore, input data include speed and flow from both detectors. The true value of average section speed is used as a third data source to validate the training accuracy of the neural network.

**Figure 1:**
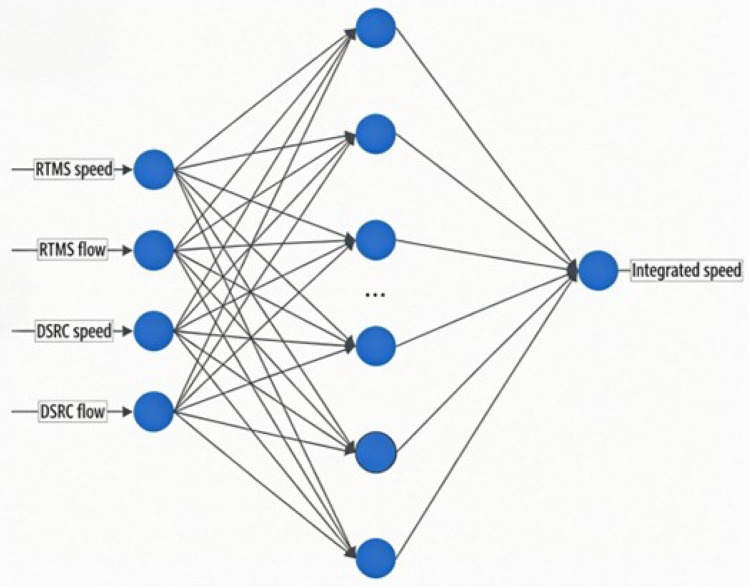
Structure of the BSO-BP neural network.

The neural network for multi-source traffic data adopts a classical three-layer structure, as shown in Figure 1. The proposed BSO-BP fusion model adopts a three-layer BP neural network architecture consisting of an input layer, one hidden layer, and an output layer. The input layer contains four neurons, corresponding to four traffic features extracted from the two data sources: RTMS flow, RTMS speed, ETC-DSRC flow, and ETC-DSRC speed. These four features represent the complementary characteristics of cross-sectional detection and interval-based trajectory detection. The hidden layer is used to learn the nonlinear mapping relationship between multi-source traffic features and the target section speed. The number of hidden-layer neurons is determined according to the empirical formula in Equation (5). The output layer contains one neuron, which represents the fused section average speed. Before BP training, the initial weights and thresholds of the network are optimized by the BAS-optimized PSO algorithm. In this process, each particle represents a candidate set of weights and thresholds of the BP neural network. The fitness value is calculated according to the prediction error of the network. After iterative optimization, the optimal particle position is used to initialize the BP neural network, and the network is further trained using the matched multi-source traffic data.

(5) $$H=\sqrt{I+O}+a$$

where:

*I*—number of input layer neurons;

*H*—number of hidden layer neurons;

*O*—number of output layer neurons;

*a*—adjustment coefficient, typically ranging from 1 to 10.

**Figure 2:**
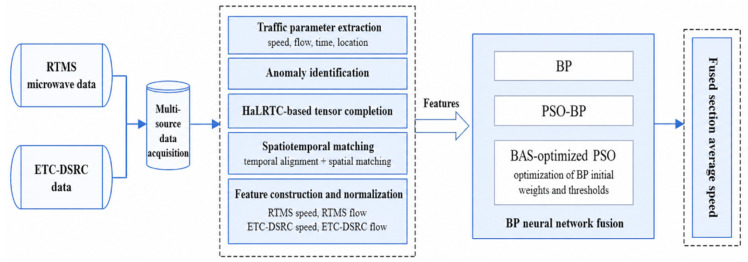
Multi-source traffic data fusion model based on the BSO-BP neural network algorithm.

The overall workflow of the proposed multi-source traffic data fusion framework is shown in Figure 2. Compared with the original BSO-BP model diagram, the revised framework explicitly includes the full data-processing and fusion procedure, including multi-source data acquisition, anomaly identification, HaLRTC-based tensor completion, spatiotemporal matching, feature construction, BSO-BP model training, and fusion output.

It should be noted that the proposed model performs feature-level multi-source traffic data fusion rather than embedding-based late fusion. ETC-DSRC and RTMS microwave data are not encoded by separate modality-specific embedding networks. Instead, after data completion, temporal alignment, and spatial matching, the traffic features extracted from the two sources are directly organized as input variables of the BSO-BP neural network. The nonlinear relationship among these heterogeneous features is then learned by the hidden layer of the BP network. Therefore, the fusion strategy in this study can be regarded as feature-level fusion implemented through a neural network, rather than late fusion of independently learned modality embeddings.

Specifically, ETC-DSRC data and RTMS microwave data are first collected and processed to extract traffic flow and speed information. Missing and abnormal values in the raw detection data are then identified and repaired using the HaLRTC tensor completion method. After data repair, temporal alignment and spatial matching are conducted to ensure that the two data sources correspond to the same road segment and time interval. The matched features, including RTMS flow, RTMS speed, ETC-DSRC flow, and ETC -DSRC speed, are normalized and used as the input of the BSO-BP neural network. In the BSO-BP model, the BAS-optimized PSO algorithm is used to determine the initial weights and thresholds of the BP neural network. Finally, the trained BP neural network outputs the fused section average speed.

## 4. Empirical Study on the Multi-Source Traffic Data Fusion Model

### 4.1. Study Section and Data Source Description

This paper conducts an empirical study on the multi-source traffic data fusion model using expressway traffic detection data, and evaluates the algorithm’s performance through specific indicators. The study section is the Jiangsu segment of the G50 Shanghai–Chongqing Expressway (also known as the Shanghai–Jiangsu–Zhejiang Expressway), which spans 49.947 km from Luyi Town (Shanghai–Jiangsu border) in the north to Zhenze Town (Jiangsu–Zhejiang border) in the south. Only the east-to-west direction (Shanghai to Jiangsu) with three lanes is considered.

As travelers perceive speed more directly and sensitively than travel time, the fusion algorithm is used to estimate average section speed to provide more accurate traffic flow information. The data sources include RTMS data from remote microwave detectors and DSRC data from mainline ETC gantries [23]. Microwave detectors record parameters such as section volume, time-mean speed, headway, and occupancy at 5-min intervals. The ETC system records real-time vehicle passage data (e.g., time, license plate) with continuous sampling. The data cover five weekdays from 18 January to 22 January 2021, across the entire G50 Expressway.

### 4.2. Processing of Fusion Data

#### 4.2.1. Spatiotemporal Matching

For the temporal dimension, microwave detector data are collected at 5-min intervals, while ETC-DSRC records are continuous. Thus, ETC-DSRC data are aggregated into 5-min bins to align timestamps with the microwave data.

**Figure 3:**
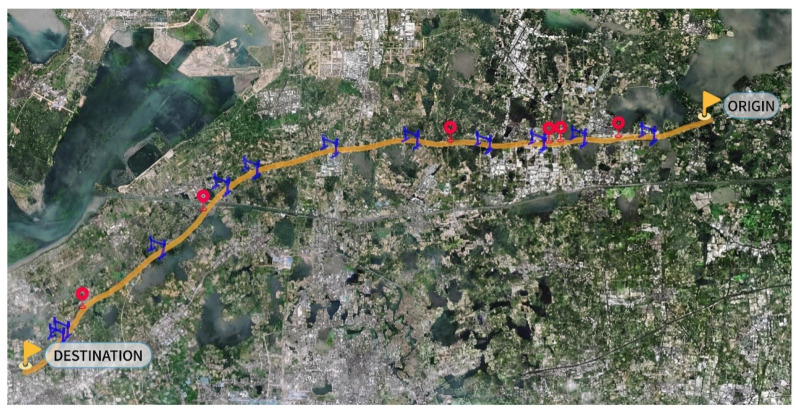
Detector deployment locations on the G50 Shanghai–Chongqing Expressway. Red circles indicate microwave detectors, and blue markers represent ETC gantries.

For the spatial dimension, both data sources include location information. Based on longitude/latitude and the milepost markers on the G50 Expressway, spatial matching is performed in a GIS system (Figure 3). The study section contains 6 microwave detectors and 11 ETC gantries, with average spacings of 8.6 km and 4.4 km, respectively. Following the method described in Section 3, the road is divided into fusion segments, as presented in Table 1.

#### 4.2.2. Data Preprocessing

1. Anomaly Data Identification

**Figure 4:**
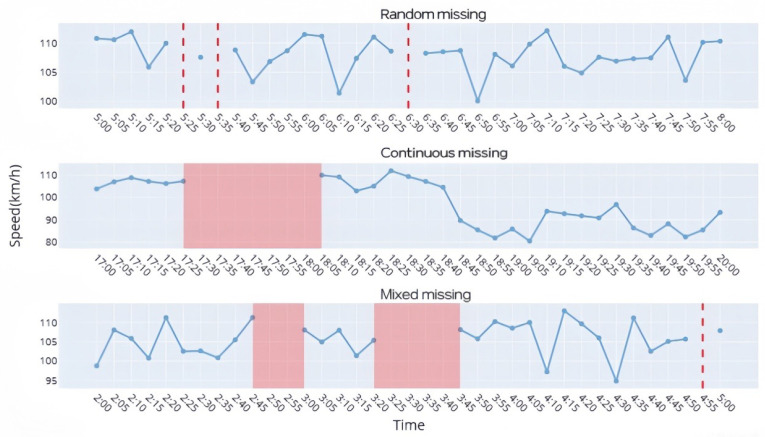
Types of missing data.

Analysis of actual traffic detection data reveals that the quality issues in microwave detection data are primarily caused by missing data. Within the spatiotemporal scope of this study, three typical types of missing data are observed, as shown in Figure 4.

(1) Random missing
  Random missing refers to cases where the data points adjacent to the missing one are relatively complete, with the missing values appearing as scattered points without correlation. The figure shows the speed distribution at section K96 + 050 between 5:00 and 8:00 on 19 January, as obtained from microwave detectors. The missing data appear as uncorrelated points.
(2) Continuous missing
  Continuous missing refers to cases where, for a missing data point, its neighboring data are also missing, forming linear patterns with correlation between consecutive missing points. This typically manifests as groups of missing values of varying lengths. The figure shows the speed distribution between 17:00 and 20:00 on 22 January, where the missing data exhibit continuity.
(3) Mixed missing
  Mixed missing combines the two previous types, with point-like and line-like missing data occurring alternately. The figure shows the speed distribution between 2:00 and 5:00 on 20 January, which represents the most common type of data missing in practice.

2. Anomaly Data Imputation

**Figure 5:**
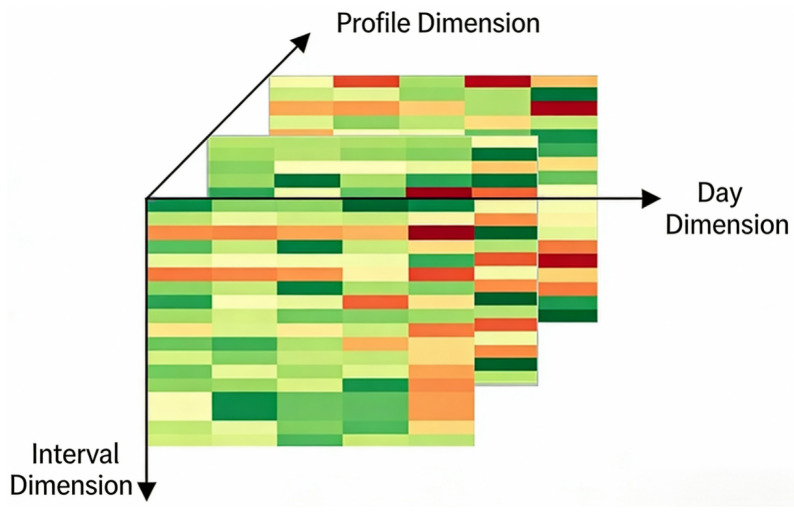
Schematic diagram of tensor model construction.

This paper constructs a multi-dimensional tensor model incorporating cross-section, day, and time period dimensions to impute traffic detection data. Detector data from three adjacent cross-sections (K76 + 200, K96 + 050, and K107 + 800) are selected, covering five consecutive weekdays from 18 January to 22 January 2021. The sampling interval is 5 min, resulting in 288 data records per day. The constructed tensor model is shown in Figure 5.

**Figure 6:**
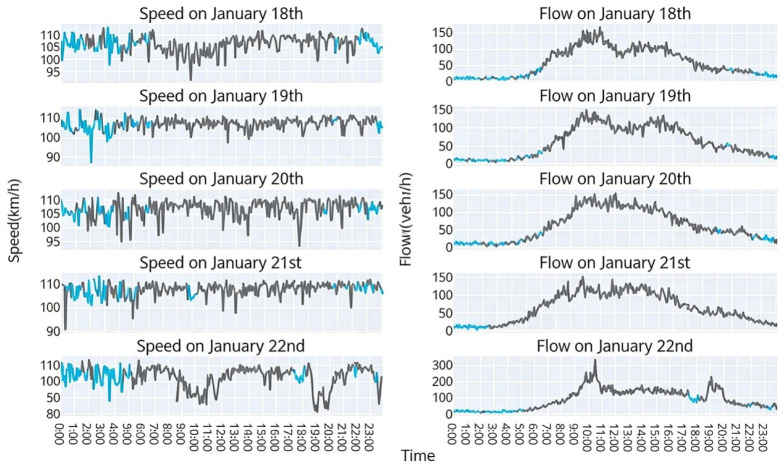
Comparison of anomalous traffic data imputation, where the gray line represents the raw data and the blue line represents the imputed data.

Using a tensor completion-based imputation method, the HaLRTC algorithm was applied to repair anomalous data in the raw detection data from 18 to 22 January for traffic volume and speed, respectively [9]. The imputed results are shown in Figure 6, where the gray line represents the raw data and the blue line represents the imputed data.

As shown in Figure 6, the imputed traffic volume and speed follow the same trend as the raw data over the week of 18–22 January, demonstrating the effectiveness of the HaLRTC algorithm for traffic data imputation. To further evaluate the imputation performance, three missing patterns (random, continuous, and mixed) were artificially generated with missing rates of 10%, 20%, 30%, 40%, and 50%. The imputation accuracy for speed data was assessed using the mean absolute percentage error (MAPE), and the results are presented in Table 2.

**Figure 7:**
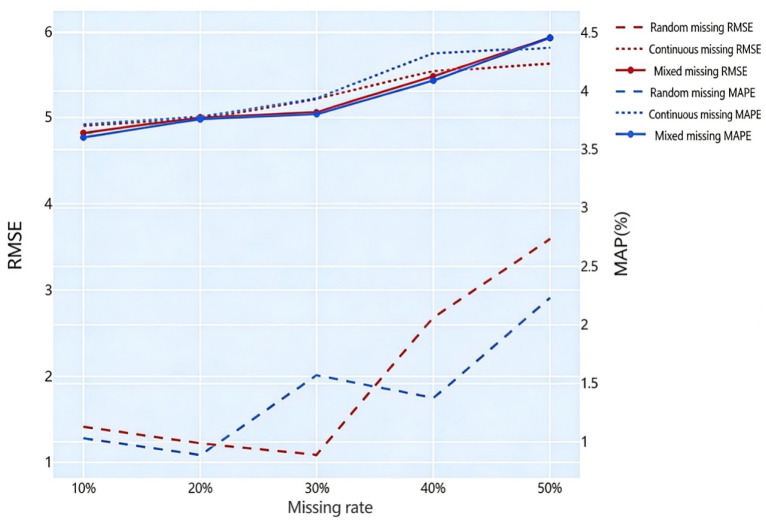
Validation of imputation performance under different missing rates.

A comparison of imputation accuracy under different missing rates is shown in Figure 7.

As shown in Figure 7, the HaLRTC imputation algorithm achieves good performance for random, continuous, and mixed missing patterns. When the missing rate is below 30%, the error for random missing is very small. Although the imputation errors for mixed and continuous missing are slightly higher than those for random missing, the maximum errors across all missing patterns are an RMSE of 5.93 and a MAPE of 4.46%, which meet the data imputation accuracy requirements of this study.

To further compare HaLRTC with commonly used imputation methods, three benchmark methods were added, including linear interpolation, K-nearest neighbor (KNN) imputation, and matrix factorization. Linear interpolation estimates missing values using adjacent temporal observations and is commonly used for short-term missing data. KNN imputation fills missing values according to the similarity between samples. Matrix factorization reconstructs missing entries by exploiting the low-rank structure of a two-dimensional data matrix. For a fair comparison, the same missing patterns, namely random missing, continuous missing, and mixed missing, were adopted for all methods. RMSE and MAPE were used as evaluation indicators.

Table 3 presents the average imputation errors of different methods under the three missing patterns. The results show that HaLRTC achieves the lowest RMSE and MAPE under all three missing patterns. Under random missing conditions, the performance differences among the methods are relatively small because adjacent temporal observations are mostly available. Nevertheless, HaLRTC still obtains the best overall performance, with an RMSE of 1.99 and a MAPE of 1.42%.

Under continuous missing conditions, the advantage of HaLRTC becomes more evident. Linear interpolation shows the largest error because consecutive missing values weaken the reliability of adjacent temporal information. KNN and matrix factorization improve the imputation performance to some extent, but their errors remain higher than those of HaLRTC. Specifically, HaLRTC reduces the RMSE to 5.25 and the MAPE to 4.02%, indicating that the tensor completion method can better recover missing values when local temporal information is insufficient.

For mixed missing conditions, HaLRTC also achieves the best performance, with an RMSE of 5.26 and a MAPE of 3.94%. This is because HaLRTC constructs traffic data as a tensor using cross-section, day, and time-period dimensions, thereby jointly capturing spatial correlations, temporal evolution patterns, and day-to-day periodicity. Compared with methods that mainly rely on adjacent observations, local sample similarity, or two-dimensional low-rank structures, HaLRTC better preserves the multi-dimensional spatiotemporal structure of traffic data and provides more robust imputation results.

### 4.3. Model Training and Evaluation

#### 4.3.1. Parameter Settings

In this section, the preprocessed traffic detection data are used to validate the multi-source traffic data fusion model based on the BSO-BP neural network algorithm. In model training, the transfer function of the hidden layer is the tansig function, the output layer uses the purelin function, and the training function is trainlm. The quadratic cost function is the mean squared error (MSE). The relevant parameter settings of the BSO-BP model are shown in Table 4:

To eliminate the influence of dimensions, the input data are standardized before training, as follows:

(6) $$x_{i}^{\prime }=\frac{x_{i}-x_{\min}}{x_{\max}-x_{\min}}$$

where:

$x_{\min}$—the minimum value in the sample;

$x_{\max}$—the maximum value in the sample.

#### 4.3.2. Model Fusion Results

**Figure 8:**
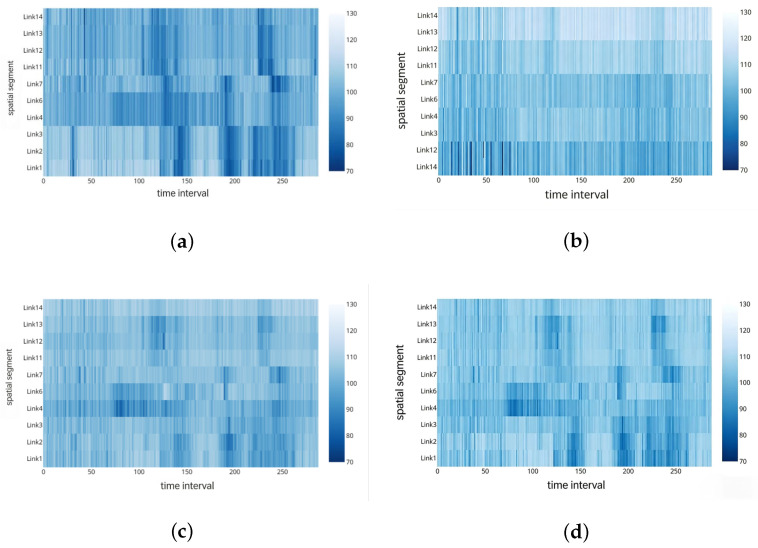
Comparison of single-source detection technologies, multi-source data fusion technology, and ground truth speed values. (**a**) RTMS single-source estimated speed values. (**b**) DSRC single-source estimated speed values. (**c**) Fused estimated speed values. (**d**) Ground truth speed values.

Based on the parameter settings determined above, the preprocessed detection data from the study section are used as experimental data to estimate the average section speed of the expressway. The fusion results are compared with those estimated from single-source detection data to demonstrate the effectiveness of the model. Data from 18 January to 21 January are used for training, while the speed data from 22 January serve as the test set. Figure 8 presents a comparison of section speeds obtained from single-source estimates and multi-source fusion techniques across each fusion segment, along with the ground truth values. In the heatmaps, color intensity reflects speed variation—darker colors indicate lower traffic speeds on the corresponding segment at that time. The dynamic changes in color allow for the reproduction of the traffic state on the segment. It can be qualitatively observed that the traffic parameters derived from the fusion estimation are significantly closer to the ground truth compared to those from single-source estimates, thereby better reflecting the actual traffic conditions [7,24].

**Figure 9:**
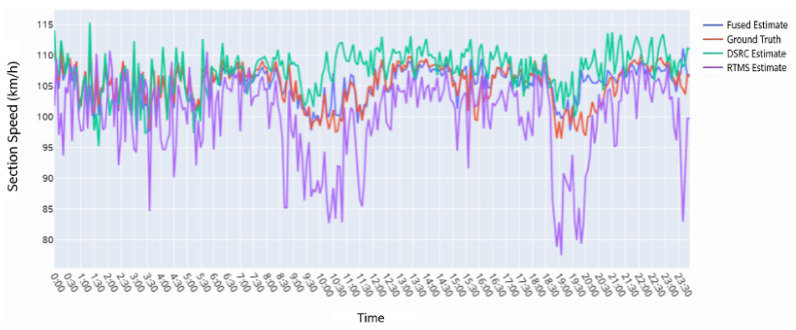
Comparison of fusion results for Link 11.

Taking Link 11 as an example, the performance of multi-source traffic data fusion is illustrated in Figure 9. During off-peak periods, the estimates from the three approaches are similar. However, during peak hours with high traffic volume and low speeds, the interval speed estimated from RTMS microwave data is lower than the ground truth, whereas the estimate derived from ETC-DSRC data is higher. This is because the RTMS-based method infers interval speed from cross-sectional spot speeds, potentially amplifying localized speed reductions to the entire segment. The DSRC-based method calculates interval speed using the time difference between two cross-sections; when ETC-DSRC gantries are widely spaced, excessive averaging can obscure localized traffic conditions. In contrast, multi-source data fusion combines both data sources, refines spatial granularity, and significantly improves the accuracy of segment-level traffic flow parameter estimation.

To quantitatively evaluate the performance of the multi-source fusion model, error metrics including mean absolute error (MAE) and root mean square error (RMSE) are introduced. Table 5 and Table 6 summarize the specific indicator values for single-source estimation and multi-source fusion estimation. It can be observed that, for all road segments, the multi-source fusion model substantially improves the estimation accuracy of traffic parameters compared with single-source estimation.

#### 4.3.3. Evaluation of the Fusion Model

**Figure 10:**
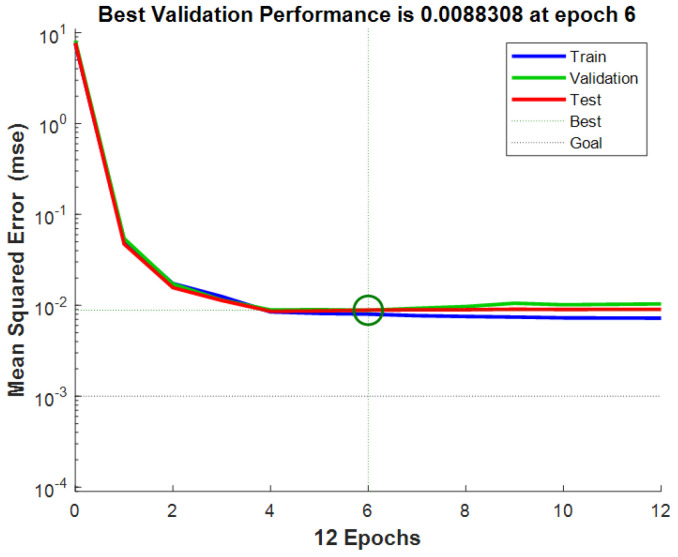
Schematic diagram of neural network training performance.

**Figure 11:**
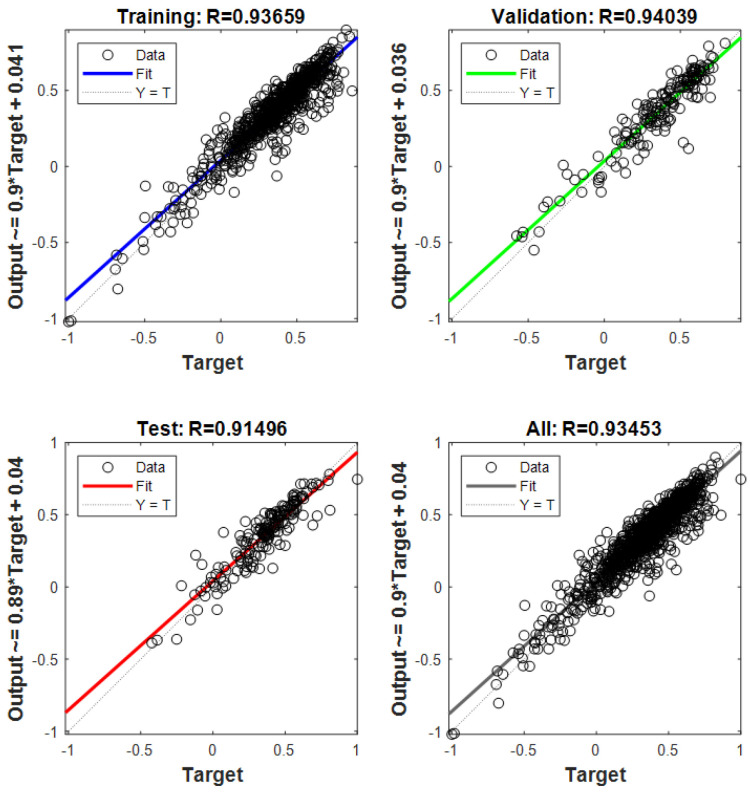
Schematic diagram of neural network fitting performance.

Figure 10 shows the training performance of the improved neural network model. After five training iterations, the mean squared error stabilizes, achieving the best validation performance with an MSE of 0.010135. During training, the data are divided into training, validation, and test sets. The fitting performance of the neural network for each subset is shown in Figure 11. Perfect fitting would align the fitting line with the diagonal, with an R value of 1. The R values for this neural network are all above 0.90, indicating good training performance.

**Figure 12:**
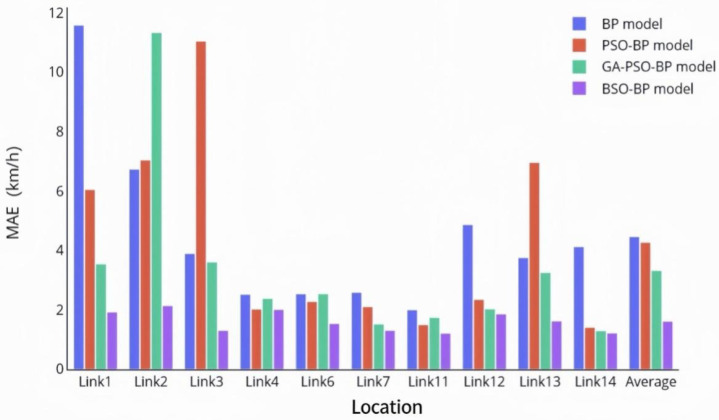
Comparison of MAE results for different models.

**Figure 13:**
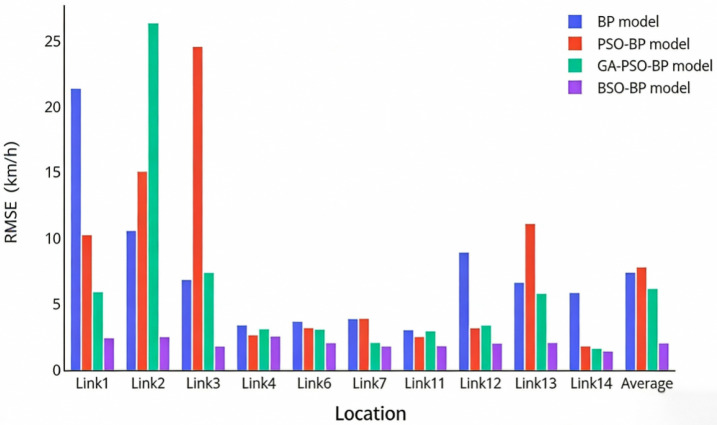
Comparison of RMSE results for different models.

In addition to the proposed BSO-BP neural network fusion model, three benchmark models, namely BP, PSO-BP, and GA-PSO-BP, were also employed for comparative evaluation under the same training and testing conditions. The purpose of this comparison is not only to verify the overall effectiveness of the proposed model, but also to examine whether the BAS-based improvement provides substantive advantages over the conventional PSO-BP model. Figure 12 and Figure 13 present the MAE and RMSE results of the four models across different road segments, respectively.

As shown in Figure 12 and Figure 13, the proposed BSO-BP model achieves lower MAE and RMSE values than BP, PSO-BP, and GA-PSO-BP across all road segments. Specifically, the average MAE and RMSE of the BSO-BP model are 1.6092 and 2.0478, respectively, which are lower than those of the benchmark models. Compared with the conventional PSO-BP model, the improvement can be attributed to the introduction of the BAS-based directional search mechanism. In PSO-BP, the particle update process mainly depends on the inertia term, the individual best position, and the global best position, which may cause particles to converge toward similar trajectories and reduce population diversity. In contrast, BSO-BP introduces an additional antennae-based search increment into the particle position update, enabling particles to explore candidate solutions from directional fitness information. This mechanism helps reduce the probability of premature convergence and improves the optimization of the initial weights and thresholds of the BP neural network.

**Figure 14:**
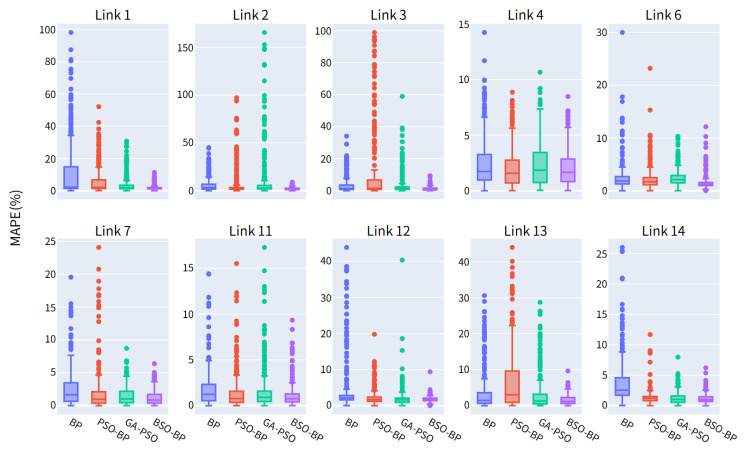
MAPE boxplot of different fusion models.

The above MAE and RMSE results reflect the overall estimation accuracy across different road segments. Since traffic conditions vary significantly over a 24-h period, especially between off-peak and peak periods, it is also necessary to analyze the distribution of individual estimation errors. Figure 14 presents the MAPE boxplots of the different models. Compared with BP, PSO-BP, and GA-PSO-BP, the BSO-BP model exhibits lower and more concentrated MAPE values, indicating that the proposed model not only improves average estimation accuracy, but also provides more stable fusion results under different traffic conditions. Therefore, the experimental results demonstrate that the BAS-based improvement brings significant advantages over the conventional PSO-BP model in multi-source expressway traffic data fusion.

## 5. Conclusions and Discussion

Based on the ETC-DSRC toll collection data and microwave detection data collected from the Jiangsu section of the G50 Shanghai–Chongqing Expressway, this paper systematically investigates multi-source traffic data extraction, preprocessing, and fusion modeling. A multi-source data fusion framework for traffic flow parameter estimation is constructed, and core conclusions are drawn through empirical validation and comparative analysis. First, to address the heterogeneity of ETC-DSRC and microwave detection data in spatiotemporal granularity, data type, and semantics, a targeted extraction process and spatiotemporal matching method are designed to achieve spatiotemporal alignment. This verifies the feasibility and necessity of multi-source traffic data fusion and provides reliable foundational data support for subsequent imputation and fusion modeling. Second, leveraging the spatiotemporal correlation of traffic flow data, the HaLRTC tensor completion algorithm is adopted for missing data imputation. This method captures global spatiotemporal features simultaneously and achieves higher accuracy under scenarios with consecutive missing values, outperforming traditional time series or historical data-based imputation methods and providing high-quality input for the fusion model. Finally, a BSO-BP neural network fusion model optimized by the beetle antennae search algorithm is proposed. By enriching population diversity and dynamically adjusting particle velocity, the convergence performance of the traditional PSO-BP algorithm is improved. Empirical results demonstrate that the proposed model outperforms single-source methods and other fusion models in traffic flow parameter estimation, fully validating the advantages of multi-source data fusion in expressway traffic state perception.

The main innovations of this study are twofold:

(1) The HaLRTC tensor completion algorithm is applied to traffic detection data preprocessing, overcoming the limitation of traditional methods that rely solely on local data and improving imputation accuracy in cases of consecutive missing data.
(2) The BSO-BP neural network fusion model is proposed to enhance the population diversity and convergence capability of the traditional PSO-BP algorithm, offering a new technical approach for multi-source traffic data fusion on expressways.

Although the empirical results demonstrate the effectiveness of the proposed framework, it should be emphasized that the current study provides a preliminary validation based on the Jiangsu section of the G50 Shanghai-Chongqing Expressway. The selected corridor contains both ETC-DSRC and RTMS microwave detection data and is suitable for verifying the feasibility of the proposed HaLRTC-based data completion method and BSO-BP-based multi-source fusion model. However, the validation scope is still limited to a specific expressway corridor, and the robustness and generalizability of the model under different road geometries, detector layouts, traffic demand patterns, and congestion conditions require further investigation. Future work will focus on expanding the validation to more road sections and different expressway corridors. Additional segments with different detector deployment densities, traffic compositions, and traffic states will be incorporated to further evaluate the transferability and stability of the proposed model. Moreover, additional data sources, such as video detection, floating car data, weather information, and traffic incident records, will be considered to enrich the multi-source fusion framework and improve the real-time traffic state perception capability for smart expressway management. 

## Figures and Tables

**Table 1:** Road segmentation results.

Segment ID	Start Location	End Location	DSRC	RTMS	Scope
Link 0	K61 + 100	K61 + 850	√	×	Shanghai–Jiangsu Mainline to Fenhu
Link 1	K61 + 850	K64 + 060	√	√	Shanghai–Jiangsu Mainline to Fenhu
Link 2	K64 + 060	K66 + 960	√	√	Fenhu to Fenhu Interchange
Link 3	K66 + 960	K68 + 980	√	√	Fenhu to Fenhu Interchange
Link 4	K68 + 980	K69 + 940	√	√	Fenhu Interchange to Beishe
Link 5	K69 + 940	K73 + 850	√	×	Fenhu Interchange to Beishe
Link 6	K73 + 850	K76 + 200	√	√	Beishe to Lili
Link 7	K76 + 200	K79 + 200	√	√	Beishe to Lili
Link 8	K79 + 200	K85 + 100	√	×	Lili to Pingwang Interchange
Link 9	K85 + 100	K91 + 000	√	×	Pingwang Interchange to Pingwang
Link 10	K91 + 000	K93 + 600	√	×	Pingwang to Hengshan
Link 11	K93 + 600	K96 + 050	√	√	Hengshan to Qidu
Link 12	K96 + 050	K100 + 100	√	√	Hengshan to Qidu

**Table 2:** Imputation errors under different missing patterns.

(a) Random Missing			(b) Continuous Missing			(c) Mixed Missing		
Missing Rate	RMSE	MAPE (%)	Missing Rate	RMSE	MAPE (%)	Missing Rate	RMSE	MAPE (%)
10%	1.4112	1.0294	10%	4.90601	3.713	10%	4.82246	3.604
20%	1.2188	0.8919	20%	4.97546	3.778	20%	4.99996	3.759
30%	1.0869	1.5680	30%	5.21736	3.935	30%	5.06032	3.801
40%	2.6808	1.3757	40%	5.53572	4.320	40%	5.47519	4.085
50%	3.5897	2.2310	50%	5.62431	4.367	50%	5.92692	4.455
Mean	1.9975	1.4192	Mean	5.2518	4.023	Mean	5.2570	3.941

**Table 3:** Comparison of imputation performance among different methods.

Missing Pattern	Metric	Linear Interpolation	KNN	Matrix Factorization	HaLRTC
Random missing	RMSE	2.81	2.45	2.15	1.99
	MAPE (%)	2.10	1.58	1.75	1.42
Continuous missing	RMSE	8.80	6.57	6.62	5.25
	MAPE (%)	6.83	5.89	5.01	4.02
Mixed missing	RMSE	8.50	7.37	6.78	5.26
	MAPE (%)	6.44	5.62	5.11	3.94

**Table 4:** Parameter settings of the BSO-BP neural network model.

Parameter	Value
Learning rate	0.01
Target accuracy	0.001
Population dimension	D=I·H+H+H·O+O
Initial step size	step=0.01·ones(D,1)
Update coefficient	c=2
Search distance	$d_{0}=step/c$
Population size	Size=40
Maximum number of iterations	$T_{\max}=200$
Cognitive coefficient	$c_{1}=2$
Social coefficient	$c_{2}=2$
Inertia weight	$w_{\max}=0.9,\;w_{\min}=0.4$
Particle position	$x_{\max}=1,\;x_{\min}=-1$
Particle velocity	$v_{\max}=0.5\cdot x_{\max},\;v_{\min}=-v_{\max}$

**Table 5:** Comparison of RMSE between single-source and multi-source estimation.

Method	Link 1	Link 2	Link 3	Link 4	Link 6	Link 7	Link 11	Link 12
RTMS	5.3062	4.2749	6.2215	5.8585	7.6707	6.1513	7.2978	6.9669
DSRC	10.2865	11.1663	4.1055	6.5688	5.4490	4.4831	4.1020	4.4799
Fusion model	2.4374	2.5127	1.7762	2.5469	2.0477	1.7750	1.8243	2.0232

**Table 6:** Comparison of MAE between single-source and multi-source estimation.

Method	Link 1	Link 2	Link 3	Link 4	Link 6	Link 7	Link 11	Link 12
RTMS	3.8020	3.5704	4.7827	4.3844	6.6050	4.9068	5.8538	6.3802
DSRC	7.3789	7.5564	3.1478	4.6013	4.2881	3.3340	3.1154	3.4613
Fusion model	1.9201	2.1335	1.2980	2.0055	1.5377	1.3025	1.2057	1.8580

## Data Availability

Data will be made available upon request.
